# Mutations in proteins involved in E-C coupling and SOCE and congenital myopathies

**DOI:** 10.1085/jgp.202213115

**Published:** 2022-08-18

**Authors:** Daniela Rossi, Maria Rosaria Catallo, Enrico Pierantozzi, Vincenzo Sorrentino

**Affiliations:** 1 Department of Molecular and Developmental Medicine, University of Siena, Siena, Italy; 2 Interdepartmental Program of Molecular Diagnosis and Pathogenetic Mechanisms of Rare Genetic Diseases, Azienda Ospedaliero Universitaria Senese, Siena, Italy

## Abstract

In skeletal muscle, Ca^2+^ necessary for muscle contraction is stored and released from the sarcoplasmic reticulum (SR), a specialized form of endoplasmic reticulum through the mechanism known as excitation–contraction (E-C) coupling. Following activation of skeletal muscle contraction by the E-C coupling mechanism, replenishment of intracellular stores requires reuptake of cytosolic Ca^2+^ into the SR by the activity of SR Ca^2+^-ATPases, but also Ca^2+^ entry from the extracellular space, through a mechanism called store-operated calcium entry (SOCE). The fine orchestration of these processes requires several proteins, including Ca^2+^ channels, Ca^2+^ sensors, and Ca^2+^ buffers, as well as the active involvement of mitochondria. Mutations in genes coding for proteins participating in E-C coupling and SOCE are causative of several myopathies characterized by a wide spectrum of clinical phenotypes, a variety of histological features, and alterations in intracellular Ca^2+^ balance. This review summarizes current knowledge on these myopathies and discusses available knowledge on the pathogenic mechanisms of disease.

## Teaser

This review provides an up-to-date overview of current knowledge on myopathies caused by mutations in proteins participating in excitation–contraction coupling and store-operated Ca^2+^ entry mechanisms in skeletal muscle.

## Introduction

Calcium ion (Ca^2+^) represents a central second messenger in eukaryotic cells, where it governs a plethora of cellular processes, including cell proliferation, secretion, and metabolism, among many others (Berridge et al., 2000). In striated and smooth muscle cells, it also plays the fundamental role of regulating muscle contraction by activating the actomyosin complex (Dulhunty, 2006). To perform all these functions, cells must accurately regulate the intracellular Ca^2+^ concentration by both controlling Ca^2+^ exchange with the extracellular environment and establishing intracellular Ca^2+^ stores for prompt utilization (Bootman and Bultynck, 2020). In skeletal muscle, most of the Ca^2+^ used during contraction cycles comes from the sarcoplasmic reticulum (SR), a specialized form of endoplasmic reticulum (ER), that forms a complex network of tubules and cisternae wrapping the myofibrils in a sleeve-like structure. The SR is composed of two distinct domains: the longitudinal SR (l-SR) and the junctional SR (j-SR). The l-SR is the main site of Ca^2+^ uptake from the sarcoplasm, thanks to the presence of sarco/endoplasmic reticulum Ca^2+^ ATPase (SERCA) pumps, while the j-SR associates with sarcolemma transverse tubules (T-tubules) to form the triads, where the ryanodine receptor type 1 (RYR1) Ca^2+^ release channels are localized (Franzini-Armstrong, 2018). Triads represent the membrane structures that support the excitation–contraction (E-C) coupling mechanism in skeletal muscle fibers, providing a site where dihydropyridine receptor (DHPR) and RYR1 can physically interact to activate Ca^2+^ release following membrane depolarization (Meissner and Lu, 1995). A third essential component of the E-C coupling machinery is the adaptor protein SH3 and cysteine-rich domain 3 (STAC3). DHPR is a voltage-dependent L-type Ca^2+^ channel located on the T-tubules that, following membrane depolarization induced by motor-neuron stimulation, undergoes a conformational change that allows the opening of RYR1. The skeletal muscle DHPR is composed of a heteromultimeric complex that includes the α1s, α2, δ, β1α, and γ1 subunits (Flucher, 2020). The α1s subunit (also referred to as Cav1.1) is an integral membrane protein containing four transmembrane domains, each composed of six α-helices, acting as the pore-forming and the voltage-sensing unit (Hu et al., 2015). Trafficking of the α1s subunit and coupling with RYR1 is regulated by STAC3 (Polster et al., 2016). Opening of RYR1 channels results in massive Ca^2+^ release from the SR into the myoplasm, which in turn triggers muscle contraction (Dulhunty, 2006). In addition to RYR1, a second isoform, RYR3, is expressed in skeletal muscle, although only RYR1 is essential for E-C coupling activation. At variance with RYR1, RYR3 channels are mainly expressed in neonatal versus adult muscles and are activated by a Ca^2+^-induced Ca^2+^-release (CICR) mechanism, independently of the interaction with DHPR (Rossi and Sorrentino, 2002).

In addition to DHPR, RYR1, and STAC3, which are essential for E-C coupling, other proteins are localized at triads and regulate this mechanism, including junctophilins (JPHs), triadin, junctin, and calsequestrin (CASQ; Rossi et al., 2022a). JPHs mediate triad assembly and maintenance by both establishing the apposition between j-SR cisternae and T-tubules and acting as a scaffold for the assembly of the Ca^2+^ release complex (Phimister et al., 2007; Golini et al., 2011; Nakada et al., 2018; Rossi et al., 2019a). In mammals, JPH1 and JPH2 are expressed in striated muscles (Takeshima et al., 2000). Triadin and junctin are transmembrane proteins composed by a cytoplasmic N-terminal domain (NTD) and a luminal C-terminal segment (Zhang et al., 1997; Dulhunty et al., 2009; Marty, 2015). They act as functional regulator of E-C coupling by interacting with RYRs (Guo and Campbell, 1995; Zhang et al, 1997; Caswell et al, 1999; Li et al, 2015; Rossi et al, 2022b), CASQ (Kobayashi et al., 2000; Shin et al., 2000; Boncompagni et al., 2012; Rossi et al., 2014; Rossi et al., 2021), and the histidine-rich Ca^2+^-binding protein (Lee et al., 2001). Triadin also plays a structural role in supporting triad architecture by interacting with the microtubule-binding protein climp-63, also known as cytoskeleton-associated protein 4 (Osseni et al., 2016). CASQ is an intraluminal SR soluble protein with high capacity and low-affinity Ca^2+^-binding properties. CASQ is the main Ca^2+^ buffering protein of the SR, although recent evidence shows that it can display a more complex regulatory role in Ca^2+^ homeostasis (see below; Beard et al., 2004; Protasi et al., 2011; Beard and Dulhunty, 2015; Manno et al., 2017; Rossi et al., 2021). Two isoforms of CASQ have been identified: CASQ1 is expressed in fast- and slow-twitch skeletal muscle fibers, whereas CASQ2 is expressed in slow-twitch skeletal muscle fibers and cardiac muscle (Biral et al., 1992).

For many years, Ca^2+^ entry from the extracellular environment was believed to play only a marginal role in muscle physiology and contraction. However, new evidence has accumulated indicating that Ca^2+^ influx through the store-operated calcium entry (SOCE) mechanism, mediated by stromal interaction molecule 1 (STIM1), a Ca^2+^ sensor in the SR, and ORAI1, a selective Ca^2+^ channel on the plasma membrane, is also fundamental to refill intracellular Ca^2+^ stores and sustain prolonged activity of skeletal muscle fibers (Launikonis and Rios, 2007; Michelucci et al., 2018). In past decades, while gaining an incredible amount of information on structural and functional aspects that support the mechanisms that govern Ca^2+^ release through the RYR1 channels, we have also recognized that mutations in genes that code for proteins involved in Ca^2+^ handling are causative of inherited myopathies. In this review, we provide an overview of the main aspects of muscle diseases linked to mutations in Ca^2+^-handling proteins in skeletal muscle, focusing on the E-C coupling and SOCE mechanisms.

## E-C coupling and congenital myopathies: *RYR1*, *CACNA1S*, and *STAC3*

**Figure 1.**
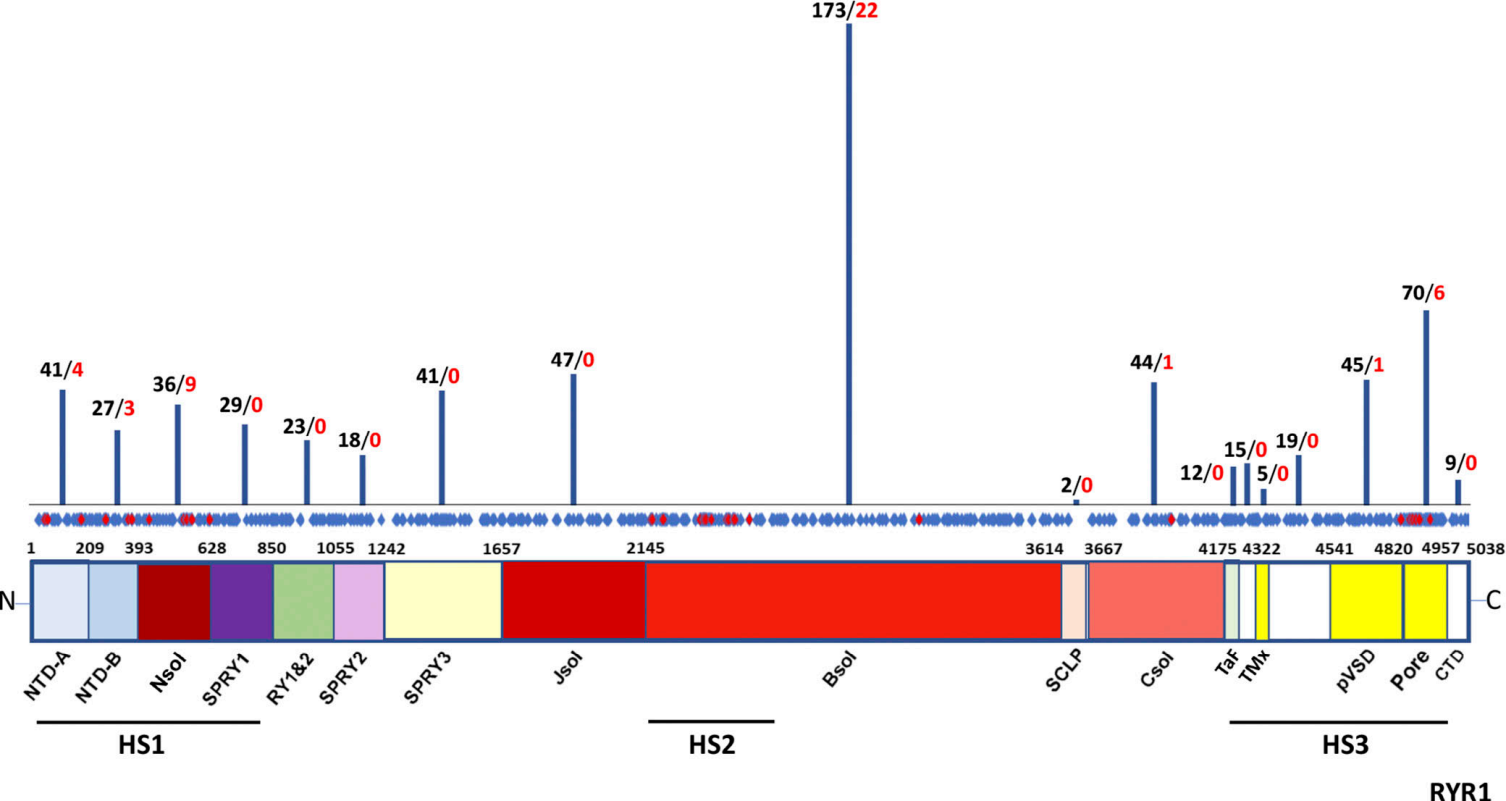
**RYR1 pathological amino acid variants aligned with corresponding RYR1 structural regions.** Black numbers on top of the bars indicate the number of different variants in the corresponding RYR1 protein domains identified in patients to date, according to Kushnir et al., 2020 and Global Variome shared LOVD (https://databases.lovd.nl/shared/variants/RYR1). Red numbers indicate diagnostic MH *RYR1* mutations in the corresponding RYR1 domain, according to the EMHG. Distribution of the different aa variants (*n* = 656, blue boxes), including MH-causative aa variants (*n* = 46, red boxes), throughout the RYR1 aa sequence is reported in the horizontal bar. RYR1 was subdivided into domains according to des Georges et al., 2016. The position of the initial aa of each domain is indicated. SCLP, shell-core linker peptide; TaF, thumb and forefingers domain; TMx, auxiliary transmembrane helices; Pore, channel pore domain.

In humans, the *RYR1* gene codes for a protein of 5,038 amino acids that assembles in tetramers of >2 MD. Structural cryo-EM studies showed that each monomer has a large N-terminal cytoplasmic region, six transmembrane domains that define the pore region, and a small cytoplasmic C-terminal portion (Efremov et al., 2015; Yan et al., 2015; Zalk et al., 2015; des Georges et al., 2016). As reported in Fig. 1, these studies showed that the cytoplasmic region is composed by an α-solenoid scaffold and some globular domains. The α-solenoid scaffold is formed by four segments, and it is capped, at the N-terminus, by two distinct NTDs, NTD-A and NTD-B; these are immediately followed by the first segment, the N-solenoid (N-sol), connected to the SP1/a ryanodine receptor domain (SPRY1) domain. This is followed by a pair of RYR repeats (RY1&2). Two other SPRY domains (SPRY2 and 3) are localized upstream of the second and the third solenoids, the junctional (J-sol) and the bridging (B-sol) solenoids, respectively, that are followed by a second pair of RYR repeats (RY3&4). Finally, the fourth and last solenoid, called the core solenoid (C-sol), precedes an EF-hand domain (Efremov et al., 2015; Yan et al., 2015; Zalk et al., 2015; des Georges et al., 2016). The high flexibility of the α-solenoid scaffold of RYR1 facilitates the interaction of the channel with regulatory proteins and molecules, such as Ca^2+^, Mg^2+^, ATP, FK-binding protein 12, or calmodulin, and contains consensus sequences for posttranslational modification by kinases and phosphatases (Lanner et al., 2010; Hernández-Ochoa et al., 2015; Yan et al., 2015; Zalk et al., 2015; des Georges et al., 2016; Woll and Van Petegem, 2022). The transmembrane pore region presents a fold shape with six transmembrane helices (S1–S6): S1–S4 form a pseudo voltage-sensor domain (pVSD), while S5 and S6 helices from the four subunits form the pore of the channel. The S6 helix extends into the cytosol, terminating in the C-terminal domain (CTD), a small α-helical domain that contains a Zn^2+^-binding domain that contacts the cytosolic region called “thumb-and-forefinger” located within the C-sol (Woll and Van Petegem, 2022).

Mutations in *RYR1* represent the most frequent cause of nondystrophic congenital muscle diseases, which are thus referred to as *RYR1*-related myopathies, and of malignant hyperthermia (MH; Kushnir et al., 2018). According to an historical view, mutations in *RYR1* were described to be clustered in three distinct hotspots, located at the NTD, in the central region, and at the CTD (Rossi and Sorrentino, 2002; Lawal et al., 2018). Nevertheless, over the years, >500 variants have been identified also outside these hotspots, although, currently, not all have been verified as causative (Kushnir et al., 2020). In Fig. 1, the distribution of all known variants identified as of today in the *RYR1* coding sequence is reported. The position of those mutations validated as causative for MH by the European Malignant Hyperthermia Group (EMHG) is shown in red. Coincidentally, nearly all the mutations validated as causative by the EMHG are within the three hotspot regions. Recent cryo-EM studies revealed that several mutations are positioned near the fourfold symmetry axis, at sites of interaction between the RYR1 subunits or at sites of interaction between domains in the same subunit, resulting in destabilization of the closed state of the channel (Tung et al., 2010; des Georges et al., 2016; Gong et al., 2021; Woll et al., 2021; Woll and Van Petegem, 2022).

From a functional point of view, pathological mechanisms due to *RYR1* mutations have been grouped into three main categories: (1) mutations causing a gain of function, (2) mutations causing a loss of function, and (3) mutations causing a reduction in RYR1 protein content (Treves et al., 2008). For convenience, in this review, mutations are listed according to their position in the human *RYR1* gene and protein.

## Gain-of-function mutations in RYR1: in vitro characterization and related mouse models

*RYR1* gain-of-function mutations can result in either hypersensitivity of the channels to physiological triggers or Ca^2+^ leak. Hypersensitivity is typically associated with MH mutations, since exposure to specific triggers, such as halogenated anesthetics, by lowering the threshold for RYR1 activation, induced a massive and uncontrolled Ca^2+^ efflux from the SR that leads to sustained muscle contraction and a hypermetabolic state (Tong et al., 1997). Among MH-causative mutations, R614C is one of the best characterized, since it is the first mutation identified in humans and is also naturally present in domestic swine populations affected by porcine stress syndrome, an MH-like crisis induced by stress, heat, or volatile anesthetics (Fujii et al., 1991; Gillard et al., 1991; MacLennan and Phillips, 1992). The R614C mutation typically confers hypersensitivity to caffeine and halothane without significantly altering the cytosolic or the luminal Ca^2+^ concentrations (Tong et al., 1997; Tong et al., 1999; Brini et al., 2005). In addition, studies performed in HEK293T cells showed that this mutation sensitizes RYR1 channels to activation by luminal Ca^2+^ by lowering the threshold for so-called store overload–induced calcium release (SOICR; Jiang et al., 2008). This mechanism was first described in cardiac muscle, where expression of some mutations in RYR2 associated with catecholaminergic polymorphic ventricular tachycardia resulted in channel opening induced by elevation of luminal Ca^2+^ (Venetucci et al., 2008). This mechanism was later confirmed in skeletal muscle for the R614C mutation, and also for other *RYR1* variants (R2163H, G2434R, R2435L, R2435H, R2454H, Y4796C, T4826I, L4838V, A4940T, G4943V, and P4973L; Chen et al., 2017).

Other mutations in *RYR1*, such as R163C or Y522S, resulted in a spontaneous and continuous Ca^2+^ leak from the SR that caused chronic elevation in resting cytosolic Ca^2+^ and reduction of the SR Ca^2+^ content (Tong et al., 1999; Chen et al., 2017). These conditions result in impaired muscle contraction, mitochondrial damage, and appearance of typical cores negative for oxidative enzyme staining, characteristic of central core disease (CCD; Loke and MacLennan, 1998). Over the years, however, the partition between mutations resulting in hypersensitive and leaky channels has become less clear-cut. As an example, low levels of spontaneous Ca^2+^ release were also observed in dyspedic myotubes expressing RYR1 channels carrying R614C, R2163C, or T4825I mutations, mainly associated with MH (Dirksen and Avila, 2002; Dirksen and Avila, 2004). Similarly, HEK293T cells expressing RYR1 channels carrying R163C, I403M, Y522S, R2163H, and R2436H mutations, typically associated with CCD, showed lower thresholds for caffeine- and halothane-induced Ca^2+^ release (Tong et al., 1997; Tong et al., 1999); this suggests the existence of a partial overlap in the functional behavior among different *RYR1* mutations that, at the clinical level, may explain the existence of mixed MH and CCD phenotypes where a fraction of patients affected by CCD may also show susceptibility to MH.

In the attempt to understand the pathogenic mechanisms leading to either MH or CCD, four knock-in mouse models were generated, with the isogenic mutations corresponding to human *RYR1* R163C (Yang et al., 2006), Y522S (Chelu et al., 2006), G2434R (Lopez et al., 2018), and T4825I (Yuen et al., 2012) mutations. All four mouse models exhibit anesthetic-triggered MH episodes and environmental heat stress, and analysis of the functional properties of mutant channels confirmed the results previously obtained using cellular models.

In detail, homozygous and heterozygous mice carrying the MH-associated mutations G2434R and T4825T are viable and display all typical hallmarks of MH, with a severity rate dependent on gene dose (homozygous > heterozygous) and sex (male > female; Yuen et al., 2012; Lopez et al., 2018). In contrast, homozygous mice for the CCD-associated mutations, e.g., R163C and Y522S, show a more severe phenotype: *Ryr1*^R163C/R163C^ mice die in utero at approximately embryonic day 17–18 (E17–18; Yang et al., 2006), and *Ryr1*^Y522S/Y522S^ mice show respiratory failure, skeletal deformities, dysmorphic muscle, likely arising from severe defects in myogenesis and bone formation, and die between E17.5 and postnatal day 1 (Chelu et al., 2006). Heterozygous mice for both R163C and Y522S show the typical hallmarks of MH upon exposure to volatile anesthetics, but unfortunately, they show a different phenotype as it concerns the formation of cores. Indeed, cores are not observed in *Ryr1*^R163C^ mice in either the homozygous or heterozygous state (Chelu et al., 2006). In contrast, *Ryr1*^Y522S/WT^ mice show structural alterations similar to those observed in humans (Boncompagni et al., 2009). Characterization of these mice depicted a hypothetical timeline for core formation that (1) starts with mitochondrial damage and formation of early cores at 2–4 mo of age; (2) evolves to formation of contracture cores that completely lack mitochondria and triads and present shortened sarcomeres; and (3) finally results in the development of unstructured cores with larger areas lacking mitochondria and contractile filaments (Boncompagni et al., 2009). The different phenotypes of these two CCD mouse models and the apparent inconsistency in mirroring the typical features of the human disease leave open the question about the nature of causative mechanisms leading to core formation. Differences between species and/or in the genetic background may be considered critical aspects.

The further characterization of these mouse models revealed that, in addition to inducing alterations in muscle contraction and/or structure, these *RYR1* mutations can impact on other aspects of muscle biology that were not previously considered. For example, extracellular Ca^2+^ entry was shown to be altered in some of these mouse models, and it was proposed that this may contribute, in the short or long term, to exacerbate muscle impairment (Yang et al., 2007; Cherednichenko et al., 2008; Bannister et al., 2010; Estève et al., 2010; Eltit et al., 2013; Yarotskyy et al., 2013; Lamboley et al., 2021). In *Ryr1*^G2434R/WT^ and *Ryr1*^T4826I/WT^ mice, the decrease in SR Ca^2+^ content due to channel leakage activates SOCE and increases mitochondrial Ca^2+^ uptake (Yang et al., 2007; Lamboley et al., 2021). This is, initially, translated into activation of a preserving mechanism that leads to increased ATP production to support the greater demand of ATP exerted by SERCA pumps in their continuous activity to counteract Ca^2+^ leak (Lamboley et al., 2021). Nevertheless, in the long term, mitochondria from both *Ryr1*^G2434R/WT^ and *Ryr1*^T4826I/WT^ mice showed structural and functional alterations, mainly in fatty acid metabolisms, that resulted in lipotoxicity and an increase in oxidative stress, impacting muscle bioenergetics and muscle performance (Yuen et al., 2012; Chang et al., 2020). Similarly, muscles from *Ryr1*^R163C/WT^ mice show an increase in excitation contraction calcium entry (ECCE) linked to delayed inactivation of L-type currents through DHPR (Cherednichenko et al., 2008; Bannister et al., 2010; Estève et al., 2010); both *Ryr1*^R163C/WT^ and *Ryr1*^Y522S/WT^ also display an increase in SOCE that may contribute to Ca^2+^ overload, hypercontractures, heat generation, and rhabdomyolysis (Eltit et al., 2013; Yarotskyy et al., 2013). Whether these alterations also occur in muscles from patients carrying the same mutations is still to be defined.

Finally, the persistent Ca^2+^ leak observed in *Ryr1*^Y522S/WT^ knock-in mice was proposed to lead to increased production of oxidative stress and reactive nitrogen species (RNS) that result in posttranslational modifications of RYR1, which in turn further enhance channel activity. In this way, a destructive feed-forward cycle is activated, leading (in acute conditions) to sudden death or inducing (over time) mitochondrial damage (Durham et al., 2008; Lanner et al., 2012; Manno et al., 2013; Canato et al., 2019). Interestingly, the use of 5-aminoimidazole-4-carboxamide ribonucleoside, a compound that, in this context, can inhibit Ca^2+^ leakage through RYR1 channels, prevented heat-induced death in *Ryr1*^Y522S^ mice (Lanner et al., 2012); furthermore, treatment with the antioxidant drug *N*-acetylcysteine significantly reduced mitochondrial damage, contributing to improved muscle function (Michelucci et al., 2017a).

## Loss-of-function mutations in RYR1: in vitro characterization and related mouse model

*RYR1* loss-of-function mutations result in reduction of Ca^2+^ release due to either an impairment of RYR1 and DHPR functional coupling, through the so called “uncoupling” mechanism (Avila et al., 2001; Avila et al., 2003), or a reduced channel opening resulting from alterations in sites of interaction with endogenous activators (Yuan et al., 2021). The majority of uncoupling mutations are localized in the C-terminal region of the channel containing the pore domain, within the ^4895^GGGIGDE^4901^ amino acid sequence in humans (Gillespie et al., 2014). The first described loss-of-function mutation in *RYR1* was I4898T, identified in patients affected by CCD (Lynch et al., 1999). Although preliminary experiments performed on heterologous channels expressed in HEK293T cells or lymphocytes from patients carrying the I4898T mutation reported an increase in SR Ca^2+^ leak (Lynch et al., 1999; Tilgen et al., 2001), further functional characterization gave opposite and surprising results. Indeed, when expressed in HEK293T cells or dyspedic myotubes, both caffeine- and voltage-induced Ca^2+^ release from homozygous channels were completely abolished, while a significant reduction in caffeine- and voltage-induced Ca^2+^ release was observed in cells expressing heterozygous channels (Avila et al., 2001; Avila et al., 2003; Xu et al., 2008). No alterations in cytosolic or SR Ca^2+^ levels were observed, suggesting that the pathogenic mechanism associated with this mutation had to be different from that proposed for mutations leading to leaky channels (Avila et al., 2003). To further characterize the *RYR1* I4898T mutation, a mouse model was generated by Zvaritch et al. (2007). Homozygous *Ryr1*^I4898T^ mice die after birth and are paralyzed due to complete lack of voltage-induced Ca^2+^ release. Skeletal muscle development is arrested at early stages of myoblast fusion, with impairment of myofibrillogenesis, resembling that observed in dyspedic mice (Takeshima et al., 1994; Zvaritch et al., 2007). Heterozygous *Ryr1*^I4898T/WT^ mice show a slow progressive congenital myopathy with variable expression of minicores, cores, and rods (Zvaritch et al., 2009), mirroring, in some way, the variable penetrance of this mutation observed in humans. It must be stated, however, that minicores or nemaline rods are not usually observed in humans carrying the *RYR1* I4898T mutation and that CCD is considered a nonprogressive disease, indicating that this model did not completely fit with the histological and clinical aspects observed in humans carrying the same mutation. A second *Ryr1*^I4898T^ mouse model generated on a different genetic background developed a mild myopathy that was much more similar to human CCD, suggesting that the genetic background plays a relevant role in disease onset and development (Boncompagni et al., 2010; Loy et al., 2011). Indeed, these mice show a preferential involvement of type I fibers that present with areas of Z line streaming and lacking mitochondria, comparable to core regions observed in type I fibers of CCD patients (Boncompagni et al., 2010).

Nevertheless, the possible mechanism of core formation in the *Ryr1*^I4898T/WT^ mouse models was not completely unraveled. An interesting point concerns the possible involvement of mitochondria in core formation: indeed, unlike *Ryr1*^Y522S/WT^, damaged mitochondria and contraction cores were only rarely observed in muscles from the *Ryr1*^I4898T/WT^ mouse model, although they were found to be displaced within a region of structural alteration; the authors could only suggest that the structural alterations observed in type I fibers may be due to long-term mechanical stress mainly affecting postural muscles (Loy et al., 2011). A third mouse model carrying the *RYR1* I4898T mutation was generated on yet another genetic background, and these mice also presented with a mild phenotype (Lee et al., 2017). The characterization of this mouse model focused on a novel possible mechanism of disease based on the observation that this mutation correlates, in these mice, with occurrence of a persistent increase in ER stress/unfolded protein response (UPR) that results in a decrease in protein synthesis and an increase in mitochondrial Ca^2+^ uptake, reactive oxygen species (ROS) production, and induction of apoptosis. According to the authors, since residue I4898 is localized in proximity of the RYR1 sequence involved in triadin binding, mutation in this residue causes a reduction in triadin binding and content. Given the interaction between triadin and CASQ, this also leads to mislocalization of CASQ, resulting in induction of ER stress/UPR. In support of this hypothesis, the use of the chemical chaperone 4-phenylbutyric acid resulted in a significant improvement of muscle function, suggesting that the ER stress/UPR pathway contributes significantly to development of CCD caused by the I4898T mutation (Lee et al., 2017).

In addition, *RYR1* mutations L4647P, F4857S, G4891R, R4893W G4899E, G4899R, A4906V R4914G, and D4918N were found to display a loss of voltage-induced Ca^2+^ release, further supporting the idea that the ^4895^GGGIGDE^4901^ amino acid sequence and sequences in close proximity to this region are required for functional E-C coupling (Monnier et al., 2001; Tilgen et al., 2001; Avila et al., 2003; Kraeva et al., 2013; Parker et al., 2017).

*RYR1* mutations that reduce channel opening by endogenous regulators such as ATP and Ca^2+^ can also be considered among loss-of-function mutations. Indeed, millimolar ATP concentrations enhance Ca^2+^-dependent activation of RYR1, resulting in increased open probability of the channels (Chan et al., 2000). Micromolar concentrations of cytosolic Ca^2+^ activate RYR1 channels, while at millimolar concentrations, Ca^2+^ acts as a channel inhibitor (Meissner et al., 1986). Indeed, the *RYR1* mutation T4980M, associated with a recessive form of congenital myopathy with cores, is located within the RYR1 ATP-binding site (Klein et al., 2011; Maggi et al., 2013). Functional studies showed that this mutation results in reduced channel activation, which may explain the muscle weakness observed in humans (Yuan et al., 2021). Similarly, the Q3969K mutation, linked to a form of CCD, is located close to the Ca^2+^ binding site of RYR1 and results in a reduction in Ca^2+^-dependent channel activation (Yuan et al., 2021).

## Mutations causing a reduction in RYR1 protein content: related mouse models

A third mechanism of disease for *RYR1*-related myopathies is associated with a decrease in the overall RYR1 protein levels; this is usually correlated with the presence of compound heterozygous mutations, where the first mutation causes a premature termination codon and the second is a missense mutation (Monnier et al., 2008; Bevilacqua et al., 2011; Cacheux et al., 2015; Brennan et al., 2019). Disease severity is linked to the nature of the mutation present in the expressed second allele as well as on the residual expression of the first hypomorphic allele (Brennan et al., 2019; Elbaz et al., 2019). Reduction in RYR1 protein content, due to compound heterozygous mutations, has been reported in several *RYR1*-related myopathies including CCD, multiminicore disease (MmD), centronuclear myopathy (CNM), congenital fiber type disproportion (CFTD), dusty core disease (DuCD), and core rod myopathy (CRM; Ogasawara and Nishino, 2021).

A mouse model mimicking the expression of compound heterozygous mutations was generated by introducing a point mutation leading to expression of the T4706M mutation in one *RYR1* allele and a 16-bp frame-shift deletion resulting in a premature stop codon in the second allele (*Ryr1*^TM/Indel^; Brennan et al., 2019). These mice show a reduction of ∼80% in RYR1 protein levels and present with muscle weakness, hindlimb paralysis, and severe scoliosis but no changes in fiber type and no evidence of cores. Mice have a short lifespan and die probably because of respiratory failure.

A second mouse model carrying a frameshift mutation (Q1970fsX16) together with the missense mutation A4329D (Ryr^Q1970fsX16/A4329D^) shows the main features of MmD-affected patients, with a reduction in muscle force and Ca^2+^ transients, associated with a decrease in RYR1 protein level of ∼65% (Elbaz et al., 2019). Interestingly, homozygous expression of the A4329D mutation (*Ryr1*^A4329D^) also shows a reduction in RYR1 protein content and muscle performance, but this was limited to slow-twitch muscle fibers. The reason for the selective effect of the homozygous A4329D mutation in slow-twitch fibers is not known; the authors suggested that a different epigenetic regulation of fast- versus slow-twitch muscle fibers may account, at least in part, for these differences (Elbaz et al., 2020). Indeed, an increase in histone deacetylase (HDAC) was observed in soleus muscle of *Ryr1*^A4329D^ mice and, interestingly, in muscles from patients carrying recessive *RYR1* mutations (Zhou et al., 2006; Rokach et al., 2015; Ruiz et al., 2022). Although the correlation between expression of mutant RYR1 channels and epigenetic changes is not clear, the use of an inhibitor of HDAC and DNA methylase significantly improved muscle strength and RYR1 expression (Ruiz et al., 2022).

Finally, a third mouse model with an inducible muscle-specific deletion of one *RYR1* allele, leading to a 50% reduction in protein expression levels, showed typical signs of a myopathy with features of CCD and DuCD, progressive muscle weakness, atrophy, and mitochondrial dysfunction (Pelletier et al., 2020). This model is particularly interesting since, unlike the previously described models, it was obtained by exclusive reduction in the expression of the *Ryr1* gene, thus excluding the existence of possible side effects due to the residual expression of mutant Ryr1 channels. In these mice, the reduction in Ryr1 protein content was sufficient to cause muscle weakness and alterations in E-C coupling. A disorganization of muscle structure was observed, with appearance of lesions resembling those observed in patients with recessive mutations, including mitochondria mislocalization and inhibition of autophagy. Interestingly, an increase in Stim1 and Orai1 was also observed, suggesting a possible role of SOCE in disease onset (Pelletier et al., 2020).

## *RYR1*-related myopathies

*RYR1*-related myopathies are classified in different subtypes mostly based on the histopathological features observed in muscle biopsies of patients (Abath Neto et al., 2017; Garibaldi et al., 2019; Knuiman et al., 2019; Lawal et al., 2020; Table 1). Nevertheless, it must be considered that the histological phenotype associated with *RYR1* mutations can differ in individuals with the same variant or change with age in the same patient, and thus classification of *RYR1*-related myopathies merely based on the histopathological pattern is more complicated than expected. In addition, MH, a nonmyopathic condition, is also strictly linked to *RYR1* mutations (Galli et al., 2006).

**Table 1.** Diagnostic cues, histological traits, and genes associated with main subtypes of E-C coupling and SOCE-related myopathies

E-C coupling–related myopathies	Main clinical features	Fiber phenotype	Causative genes	Inheritance	Mechanism (RyR1 channel)
CCD	✓ Infantile nonprogressive hypotonia and motor development delay ✓ Mild proximal muscle weakness ✓ Respiratory distress ✓ High arched palate ✓ Craniofacial dysmorphism	✓ Centrally located, well-demarcated cores, spanning the whole fiber axis ✓ Predominance in type 1 fibers ✓ Increased central nuclei	*RYR1* >90%	AD or AR	GoF, LoF
			*MYH7*	AD	Altered assembly and function of myosin dimers
MmD	✓ Axial muscle weakness, scoliosis, respiratory insufficiency, and limb joint hyperlaxity ✓ Ophthalmoplegia ✓ Arthrogryposis ✓ Hand amyotrophy	✓ Numerous cores in a limited area on longitudinal section ✓ Multiple internally located nuclei ✓ Predominance in type 1 fibers	*RYR1* ∼20% (homozygosity or compound heterozygosity)	AR	GoF, LoF, lower protein levels
			*SEPN1* ∼50%	AR	Altered redox activity
			*TTN* (homozygosity or compound heterozygosity)	AR	M-line alteration
			*MYH7*	AD	Not defined
			*ACTA1*	AR	Not defined
			*MEGF10*	AD or AR	Not defined
			*CACNA1S*	AD	Lower protein levels
CNM	✓ Not progressive proximal muscle weakness ✓ Not progressive hypotonia	✓ Centralized and internalized nuclei ✓ Peripheral halos depleted of oxidative activity ✓ Cores	*RYR1* ∼15% (compound heterozygosity)	AR	Lower protein levels
			*MTM1*	XLR	Altered vesicle trafficking
			*DNM2*	AD	Altered membrane fission
			*BIN1*	AD	Altered membrane tubulation
			*TTN*	AR	M-line alterations
			*SPEG*	AR	Altered interaction with MTM1 and desmin
CFTD	✓ Static or slowly progressive muscle weakness ✓ Respiratory and proximal axial weakness ✓ Ophthalmoplegia ✓ Dysphagia ✓ Facial muscle weakness	✓ Fiber size disproportion (35–40% of type 1 fibers are smaller in size than type 2 fibers) ✓ Age-related development of rods, cores, and central nuclei	*RYR1* ∼20%	AR	Lower protein levels
			*ACTA1*	AD	Altered interaction with TPM
			*TPM2*	AD or AR	Altered interaction with actin
			*TPM3*	AD or AR	Altered interaction with actin
			*SEPN1*	AR	Altered redox activity
			*MYH7*	AD	LoF, altered interaction with myosin binding protein
			*LMNA*	AD	Not defined
			*ZAK*	AR	LoF
			*SPEG*	AR	LoF
DuCD	✓ Ocular involvement (eyelid ptosis, ophthalmoplegia)	✓ Irregularly sized/shaped “dusty” cores (reddish-purple granular material deposition) spanning 10–50 sarcomeres ✓ Myofibrillar disorganization	*RYR1*	AR	Lower protein levels
CRM	✓ Nonspecific clinical features, including hypotonia, muscle weakness, scoliosis, and respiratory insufficiency	✓ Nemaline bodies (rods), clustered or widely distributed along the fibers ✓ Central cores	*RYR1*	AD or AR	GoF, LoF
			*CFL2*	AR	Protein misfolding and degradation
			*ACTA1*	AD	Altered stability or function
			*TPM3*	AD or AR	Not defined
			*NEB*	AR	Not defined
MH	✓ Muscle rigidity and cardiac arrhythmia, occurring only following exposure to succinylcholine and volatile anesthetics ✓ Sustained contractures, ✓ Hyperthermia ✓ Hyperkalemia ✓ Hypermetabolism	✓ No histological features can be found in muscle fibers from MH patients	*RYR1*	AD	GoF
			*CACNA1S*	AD	GoF
**SOCE-related myopathies**					
TAM/Stormorken syndrome	✓ Muscle weakness ✓ Myalgia ✓ Cramps ✓ Increased creatine kinase levels ✓ Exercise intolerance	✓ Single- or double-walled SR tubules arranged as honeycomb-like structures in type 2 fibers ✓ Prevalence of type 1 fibers	*STIM1*	AD	GoF
			*ORAI*	AD	GoF
			*CASQ1*	AD	Altered polymerization
			*RYR1*	AD	GoF

Proteins encoded by the indicated genes and relative references are reported in the text. AD, autosomal dominant; AR, autosomal recessive; GoF, gain-of-function; LoF, loss-of-function; XLR, X-linked recessive.

### CCD

Core myopathies represent a heterogeneous group of muscle diseases that can differ clinically, pathologically, and genetically; at histological examination, they share a common pathological feature consisting of areas of muscle fibers, called cores, that do not show oxidative enzyme staining because of a reduced number of mitochondria or decreased oxidative enzyme activity. Accordingly, CCD is characterized, at the histopathological level, by centrally located and well-demarcated cores, presenting with reduced oxidative activity, running almost along the entire axis of the fibers, and mostly present in type 1 fibers (Jungbluth, 2007; Jungbluth et al., 2018; Lawal et al., 2020). Cores can be classified as structured or unstructured based on ATPase activity levels and myofibrillar disruption. Additional histopathological features of CCD include increased central nuclei, endomysial fibrosis, and proliferation of sarcotubular membranes (Garibaldi et al., 2019; Lawal et al., 2020). These cores may also contain structural proteins such as desmin, αβ-crystallin, or SR proteins such as RYR1, triadin, and DHPR (Ogasawara et al., 2020). CCD is the most frequent core myopathy, and *RYR1* mutations are found in >90% of patients (Wu et al., 2006; Jungbluth et al., 2018; Lawal et al., 2020).

Although in *RYR1*-related myopathies little or no correlation can be found between the clinical phenotype and localization of mutations in the RYR1 channel, mutations in patients with CCD are more frequently localized in the C-terminal region, containing the pore-forming domain of the channel (Wu et al., 2006). CCD can be inherited with a dominant or recessive transmission. CCD due to dominant *RYR1* mutations is the most frequent and is usually associated with a mild condition compared with the more severe cases due to recessive inheritance. Clinically, CCD has a typical onset in infancy or early childhood; patients show nonprogressive hypotonia and motor development delay, congenital dislocation of the hips, scoliosis, myalgia, and muscle stiffness; respiratory, bulbar, and cardiac involvement are less common (Bönneman et al., 2014; Lawal et al., 2018; Jungbluth et al., 2018). Serum creatine kinase levels may be moderately elevated or normal (Klein et al., 2011). CCD due to recessively inherited *RYR1* mutations presents more severe features such as marked hypotonia, multiple arthrogryposis, and respiratory failure (Bharucha-Goebel et al., 2013). Some, but not all, CCD patients carrying dominant *RYR1* mutations may have an increased risk for MH (Rosenberg et al., 2015). On the other hand, *RYR1* mutations found in patients with CCD may be present in individuals with MH susceptibility but no sign of myopathy.

As previously discussed, CCD is associated with both gain of function and loss of function. To unravel the pathogenic mechanisms of core formation due to such functionally different *RYR1* mutations, three mouse models of CCD have been generated in the last 15 yr, *Ryr1*^R163C^, *Ryr1*^Y522S^, and *Ryr1*^I4898T^ mouse models (Chelu et al., 2006; Yang et al., 2006; Zvaritch et al., 2007; Loy et al., 2011; Lee et al., 2017). A model for core formation in CCD murine models proposes that persistent Ca^2+^ leak from Ryr1 mutant channels induces a chronic condition of ROS/RNS stress, leading to mitochondrial damage, disruption of the sarcotubular system, and thus core formation. These pathogenic mechanisms are further exacerbated by the activation of futile cycles powered by ROS/RNS-dependent Ryr1 hyperactivation and increased SOCE to compensate for Ca^2+^ leak. As concerns core formation in *Ryr1*^I4898T^ mice carrying a *RYR1* uncoupling mutation, it is clear that core formation is unlikely to be due to increased intracellular Ca^2+^. Studies by Lee et al. (2017) proposed that this *RYR1* mutation induces a chronic condition of ER stress/UPR that results in a decrease in protein synthesis and an increase in mitochondrial Ca^2+^ uptake, ROS production, and induction of apoptosis. According to these models, it can therefore be hypothesized that, although the primary trigger is different, core formation in CCD, due to gain-of-function or loss-of-function mutations in *RYR1*, can represent the final outcome of the stressful conditions that converge into alterations in activity and/or structure of mitochondria and disruption of SR and myofibrils.

Dominant mutations in the *MYH7* gene, which encodes the slow/β-cardiac myosin heavy chain (MyHCI) expressed in type 1 muscle fibers and in the heart, are causative of ∼10% of CCD cases (Fananapazir et al., 1993; Romero et al., 2014), as well as of several other myopathies including MmD and CFTD (Tajsharghi et al., 2003; Cullup et al., 2012; Clarke et al., 2013). *MYH7* mutations in skeletal myopathies are usually located in the rod domain of the protein (Tajsharghi and Oldfors, 2013; Fiorillo et al., 2016). Although the pathogenic mechanisms associated with mutation in *MYH7* are still unclear, mutations in the rod region can affect assembly of functional myosin dimers or their incorporation into thick filaments, causing aberrant accumulation of myosin as in myosin storage myopathy (Tajsharghi and Oldfors, 2013).

### MmD

Recessive mutations in *RYR1* represent the second genetic cause of MmD, a myopathy presenting numerous cores, visible as pale spots in oxidative stained muscle sections, sometimes with a moth-eaten appearance and gathered in a limited area on longitudinal section. Multiple internally located nuclei, minimal myofibril disruption, and type 1 fiber predominance are noted in affected muscles, although the histological traits can be extremely variable (Lawal et al., 2018; Ogasawara and Nishino, 2021). Clinical features of MmD are variable and can be classified in four groups: (1) the classic form, with axial muscle weakness, scoliosis, respiratory insufficiency, and limb joint hyperlaxity; (2) the ophthalmoplegia form; (3) the early-onset form with arthrogryposis, and (4) a slowly progressive form with hand amyotrophy (Jungbluth et al., 2005; Jungbluth, 2007; Monnier et al., 2008; Treves et al., 2008; Klein et al., 2012; Romero and Clarke, 2013). MmD patients carrying mutations in *RYR1* typically present with extraocular muscle involvement and ophthalmoplegia, symptoms not observed in patients with mutations in *SEPN1*, which are present in the majority of MmD cases (Villar-Quiles et al., 2020)*. SEPN1* codes for selenoprotein-N, an SR protein with Ca^2+^-dependent redox activity (Ferreiro et al., 2002; Petit et al., 2003; Chernorudskiy et al., 2020; Villar-Quiles et al., 2020). The pathogenic mechanisms of RYR1-related MmD are variable, including both loss-of-function and gain-of-function mutations, as well as reduction in RYR1 protein content (Jungbluth, 2007).

More rarely, patients with MmD may carry mutations in *TTN*, *MYH7*, *ACTA1*, *MEGF10*, and *CACNA1S.* Homozygous and compound heterozygous mutations in the *TTN* gene, which codes for titin, a giant sarcomeric protein, are found in patients with MmD (Chauveau et al., 2014). These mutations are preferentially found in the region of *TTN* coding for the M-line segment of this protein (Chauveau et al, 2014; Ávila-Polo et al, 2018). Mutations in *ACTA1*, which codes for skeletal muscle α-actin, the principal isoform found in the adult sarcomeres, are rarely found in patients with MmD as well as several other myopathies (Sparrow et al., 2003). The pathogenic mechanisms of disease remain to be identified. The *MEGF10* gene encodes the multiple EGF-like domain 10 protein, a transmembrane receptor belonging to the multiple epidermal growth factor–like domains family, that is upregulated in activated satellite cells and regulates the progression of the myogenic program (Holterman et al., 2007). Mutations in *MEGF10* have been detected in a few cases of MmD (Boyden et al., 2012; Liewluck et al., 2016; Takayama et al., 2016; AlMuhaizea et al., 2021). The *CACNA1S* gene encodes Cav1.1, the pore-forming subunit of the skeletal muscle voltage-gated Ca^2+^ channel (DHPR). Recessive and dominant mutations in *CACNA1S* were identified in patients showing MmD/CNM with structural alterations in T-tubules and SR. Although the pathogenetic mechanism has not been clearly defined, both recessive and dominant mutations correlate with a strong decrease in protein levels, suggesting instability or degradation of DHPR complexes. RYR1-mediated Ca^2+^ release was also impaired, although no change in cytosolic or SR Ca^2+^ levels was observed (Schartner et al., 2017).

### CNM

Recessive mutations in *RYR1* are the most common cause of autosomal recessive CNM, accounting for ∼15% of patients. At clinical presentation, autosomal CNM most frequently shows delayed motor milestones, nonprogressive muscle weakness and hypotonia, ptosis, ophthalmoplegia, and mild to severe respiratory impairment (Jungbluth et al., 2008; Wilmshurst et al., 2010; Bevilacqua et al., 2011; Romero and Bitoun, 2011; Gòmez-Oca et al., 2021). From a histological point of view, CNM shows centralized and internalized nuclei, peripheral halos depleted of oxidative activity, and cores, although based on which gene is mutated, the histological patterns may vary (Nicot et al., 2007; Bevilacqua et al., 2011; Gomez-Oca et al., 2021). *RYR1*-related CNM often presents as a mixed phenotype between CNM and core myopathies, with the presence of multiple central nuclei and core-like structures with no limited boundaries (Abath Neto et al., 2017). These forms are normally caused by compound heterozygous mutations in *RYR1*, where one of the two mutations usually cause premature termination of the transcript, resulting in a decrease in RYR1 protein expression levels (Gòmez-Oca et al., 2021). Mutations in *MTM1* account for >50% of CNM cases. This gene encodes the 3′-phosphoinositide phosphatase myotubularin (Hnia et al., 2012; Vandersmissen et al., 2018). Mutations in the gene encoding Dynamin 2 (*DNM2)* are the most common cause of dominant CNM. DNM2 is a GTPase protein that can bind and organize the microtubular and actin cytoskeleton and associates with nascent vesicles to induce their release (Ferguson and De Camilli, 2012). More rare cases of CNM are due to mutations in *BIN1*, which encodes amphiphysin 2, a protein implicated in membrane curvature, tubulation, and vesicle trafficking (Peter et al., 2004). *MTM1*, *BIN1*, and *DNM2* encoded proteins are functionally interconnected, since they are involved in vesicle trafficking, membrane fission, or autophagy (Durieux et al., 2012). MTM1 recruits BIN1 and DNM2 to muscle membranes and enhances BIN1 tubulation. Mutations in *MTM1* affect triad structure (Royer et al., 2013; Cowling et al., 2017). Given the functional interplay between BIN1, DNM2, and MTM1, a correct balance in their reciprocal expression levels has been shown to be important for muscle physiology. Indeed, overexpression of BIN1 in *Dnm2*^R465W/WT^ and *Dnm2*^R465W/R465W^ mice improved muscle atrophy and rescued the perinatal lethality and survival of Dnm2^R465W/R465W^ mice (Lionello et al., 2022). Moreover, the use of antisense oligonucleotides against *Dnm2* in *Bin1* knockout mice improved muscle force and intracellular architecture (Silva-Rojas et al., 2022). Recently, the role of a microRNA identified within the genomic sequence of DNM2, namely miR-199a-1, was also investigated as a player in the development of CNM (Chen et al., 2020). Recessive *TTN* mutations result in a variable histological pattern, from cores to typical minicores with a marked type 1 fiber predominance. Multiple centrally located nuclei are also present in a significant proportion, thus accounting for the diagnosis of CNM (Ceyhan-Birsoy et al., 2013). Recessive mutations in the *SPEG* gene, which encodes the striated muscle enriched protein kinase, a serine/threonine kinase member of the myosin light chain kinases involved in muscle development (Luo et al., 2021), were identified in patients with severe pediatric forms of CNM with cardiac involvement (Agrawal et al., 2014; Wang et al., 2017; Tang et al., 2020).

### CFTD

CFTD diagnosis is based on the observation that 35–40% of type 1 fibers show a smaller size than type 2 fibers, without other structural defects (Clarke, 2011). However, many patients with age may develop rods, cores, and central nuclei (Garibaldi et al., 2019; Lawal et al., 2020). Recessive mutations in *RYR1* are found in ∼20% of CFTD patients, while another 40% of cases are associated with mutations in different genes including *ACTA1*, *TPM3*, *TPM2*, *SEPN1*, *MYH7*,* LMNA*, and *ZAK*; the remaining 40% of CTFD cases have not yet been associated with a genetic cause (Laing et al., 2004; Laing et al., 2009; Lawlor et al., 2010; Ortolano et al., 2011; Kajino et al., 2014; Vasli et al., 2017; Moreno et al., 2020). Clinically, the disease shows static or slowly progressive generalized muscle weakness from infancy as well as respiratory and proximal axial muscle weakness, multiple joint contractures, scoliosis, long thin face, and high arched palate. About 30% of patients also show respiratory involvement. Other common features are ophthalmoplegia, dysphagia, and facial muscle weakness (Clarke, 2011; Lawal et al., 2020). Mutations in *ACTA1* account for ∼10% of CFTD cases, are often localized on the surface of ACTA1, and correspond to amino acids that are exposed on the F-actin filament facing the sites of interaction with tropomyosin, suggesting that the mechanism of disease may correlate with disruption or alteration of the actin–tropomyosin interaction (Laing et al., 2004; Laing et al., 2009; Matsumoto et al., 2022). Cardiomyopathy is a rare finding accompanying CFTD with ACTA1 mutation (Laing et al., 2009; Matsumoto et al., 2022). *Tropomyosin 2* (*TPM2*) and *TPM3* encode two isoforms of tropomyosin expressed in skeletal muscles. *TPM2* codes for β-tropomyosin expressed in type 1 muscle fibers and to a lesser extent in fast-twitch fibers, while *TPM3* codes for slow α-tropomyosin expressed exclusively in slow-twitch fiber types (Marttila et al., 2014). Dominant mutations in *TPM2* and *TPM3* are more frequently associated with CFTD, while recessive mutations are rare (Lawlor et al., 2010; Clarke, 2011; Moreno et al., 2020). Most *TPM2* mutations are localized in the coiled-coil domain of the protein; the R133W mutation in *TPM2* alters tropomyosin flexibility and disrupts the actin–tropomyosin and actin–myosin interactions (Borovikov et al., 2020). In contrast, mutations in *TPM3* are mainly localized in the actin-binding domain of the protein, resulting in either hyper- or hypocontractility (Yuen et al., 2015).

The *LMNA* gene codes for lamin A and C proteins, structural components of the nuclear lamina, a network underlying the inner nuclear membrane (Fisher et al., 1986). A dominant mutation in *LMNA* was identified in patients with CFTD who show muscle weakness, hypotonia, and cardiac involvement (Kajino et al., 2014). Mutations in *MYH7* were rarely identified in CFTD. A frameshift mutation was proposed to disrupt the stability of the myosin rod domain and introduce a proline residue that may alter chain flexibility and dimerization. Other distal mutations in *MYH7* were also identified in a myopathy with predominance of small type I fibers (Muelas et al., 2010; Ortolano et al., 2011; Clarke et al., 2013; Pajusalo et al., 2016). Patients with recessive mutations in the *ZAK* gene, encoding the mitogen-activated protein triple kinase ZAK, were identified in three unrelated families diagnosed with CFTD who presented with slowly progressive muscle weakness, developmental delay, and scoliosis (Vasli et al., 2017). All mutations resulted in loss of protein expression due to the presence of premature stop codons in the mRNA. ZAK is a serine-threonine kinase activating the ERK, JNK, and p38 pathways, that, among other functions, regulates myogenesis (Gotoh et al., 2001). Recently, a homozygous mutation in the C-terminal region of *SPEG* has been described in patients with a mild form of CFTD with severe cardiac involvement, but in whom hypotrophic type I fibers were not observed. The mutation results in the appearance of a premature stop codon in the C-terminal region of *SPEG*. This mutation does not affect the domain of interaction with MTM1, however, and this may partially explain the mild phenotype of patients (Gurgel-Giannetti et al., 2021).

### DuCD

DuCD is defined by irregular areas of myofibrillar disorganization with reddish-purple granular material depositions, devoid of ATPase activity (Bevilacqua et al., 2011). Dusty cores are irregular in size and shape, with no demarked borders, sometime with a star-like appearance (Garibaldi et al., 2019). Unlike CCD, dusty cores are mostly 10–50 sarcomeres in length, with strands of osmophilic material accumulated in specific regions and containing SR or cytoplasmic structures. Patients with DuCD show early disease onset and severe clinical phenotype, with ocular involvement in most of the cases. DuCD is caused by recessive, biallelic *RYR1* mutations, resulting in low levels of RYR1 expression. It has been speculated that severe *RYR1* haploinsufficiency may impair integrity of triads, which are often duplicated in patients’ biopsies (Garibaldi et al., 2019).

### CRM

As its name suggests, muscles from patients affected by CRM show both central cores and nemaline bodies (rods) that are typical of nemaline myopathy. Rods are mainly composed of actin and α-actinin, probably deriving from Z disks; they can be assembled in clusters or widely distributed along the fibers (Scacheri et al., 2000). Longitudinal sections may also show cores devoid of mitochondria covering a large part of the fiber axis. From a clinical point of view, nonspecific clinical features such as hypotonia, muscle weakness, scoliosis, and respiratory insufficiency are observed. Dominant mutations in *RYR1* are the main genetic cause of CRM (Monnier et al., 2000; von der Hagen et al., 2008; Hernandez-Lain et al., 2011). Compound heterozygous mutations in *RYR1* were identified in a patient with fetal akinesia, hypotonia, ophthalmoplegia, and respiratory insufficiency (Kondo et al., 2012).

Other causative genes in CRM have been reported (*CFL2*, *ACTA1*, and *TPM3*), although some of them are typically associated with nemaline myopathy, where cores are not present (Agrawal et al., 2007; Lawal et al., 2018; Pinto et al., 2019). Mutations in cofilin 2 (*CFL2*), coding for the actin-binding protein cofilin-2, were identified in patients with nemaline myopathy with minicores and concentric laminated bodies (Agrawal et al., 2007). Dominant mutations in *ACTA1* and *TPM3* are most frequently associated with nemaline myopathy characterized by early onset and respiratory insufficiency, while recessive forms of nemaline myopathy are most frequently associated with mutations in the nebulin gene (*NEB*). Mutations in *ACTA1* are distributed along the entire gene, affecting stability, function, and conformation of the protein, so a clear correlation with genetic defect and disease onset and development is difficult to discern (Sparrow et al., 2003). Finally, a compound heterozygous mutation in *RYR3* coding for the second isoform of RYR expressed in skeletal muscle was identified in a family with nemaline myopathy. Unfortunately, RYR3 mutant channels were not characterized from a functional point of view, and thus no pathogenetic mechanism can be hypothesized (Nilipour et al., 2018).

### MH

MH is a pharmacogenetic disorder that results in a hypermetabolic state following exposure to succinylcholine and volatile anesthetics such as halothane, sevoflurane, desflurane, and isoflurane. These may cause a massive Ca^2+^ release in skeletal muscles, resulting in variable clinical manifestations that can be graded based on symptoms (Larach et al., 1994; Larach et al., 2010). This condition is potentially lethal if not rapidly treated with the muscle relaxant dantrolene (Hopkins, 2011; Ellinas and Albrecht, 2020). The incidence of MH is estimated at ∼1:100,000 individuals, although the number of people carrying mutations in *RYR1* may be estimated at ∼1:2,000–3,000 (Monnier et al., 2001; Riazi et al., 2018).

MH appears to have a higher prevalence in males than females, likely because of smaller muscle mass and a protective effect of estrogens in the latter (Michelucci et al., 2017b). Although ∼10% of patients present with no alteration at histological analysis, the remaining 90% show a variable range of alterations, including an increase in fiber size variability, internal nuclei, type 1 fiber predominance, and loss of oxidative stain that is rarely associated with the presence of cores and rods (Knuiman et al., 2019). These histological alterations, although variable, may represent mild features that, in *RYR1*-related myopathies, evolve to more severe histological and structural alterations. The diagnostic test for MH susceptibility is an in vitro contracture test, which determines the contracture threshold for skeletal muscle bundles treated with caffeine and halothane (Allen et al., 1998; Hopkins, 2011; Hopkins et al., 2015). *RYR1* variants account for ∼76% of MH events, while only 1% are linked to *CACNA1S* mutations and <1% are linked to mutations in *STAC3* (Johnston et al., 2021). As of today, 48 mutations in *RYR1* and 2 mutations in *CACNA1S* are considered causative for MH according to the EMHG, since they have been characterized at the genetic and functional level (Hopkins et al., 2015). Each of these mutations has been fully characterized at the genetic level, including aspects concerning evolutionary conservation and change in charge, polarity, or structure introduced by the amino acid replacement, cosegregation of the variant with the disease in the families affected, and assessment of the prevalence of the variant in the population. In addition, each mutation has been functionally characterized using one or a combination of test systems including expression of recombinant proteins in muscle or nonmuscle cells or patient-derived myotubes and lymphoblasts (Hopkins et al., 2015). However, hundreds of additional variants in *RYR1* have been identified in genetic studies of MH-susceptible individuals, although functional characterization is not yet available for all (Kushnir et al., 2020). Recently, to help in classifying genetic variants in *RYR1*, a score matrix has been defined based on the identification of pathogenic or benign criteria of the American College of Medical Genetics and Genomics (Johnston et al., 2021).

A small percentage of individuals carrying *RYR1* mutations can experience MH-like episodes independently of anesthesia, a condition also referred to as awake MH, which is characterized by skeletal muscle cramping and rigidity, rhabdomyolysis associated with exertional heat illness (the inability to thermoregulate during physical activity), exertional rhabdomyolysis, emotional stress, fatigue, and viral infection (Tobin et al., 2001; Wappler et al., 2001; Capacchione and Muldoon, 2009; Sambuughin et al., 2009; Groom et al., 2011; Dlamini et al., 2013; Molenaar et al., 2014; Timmins et al., 2015; Sambuughin et al., 2018; Zvaritch et al., 2019; Gardner et al., 2020; Laitano et al., 2020; Kruijt et al., 2022). Although the mechanisms that trigger awake MH have not yet been defined, the correlation with anesthetic-induced MH is supported by studies in animal models that include pigs carrying the R615C mutation corresponding to the human R614C mutation (Fujii et al., 1991) and mice carrying *RYR1* mutations equivalent to human Y522S or G2434R mutations, which show susceptibility to undergo MH crisis when exposed to environmental stress (Michelucci et al., 2017b; Michelucci et al., 2017c; Lopez et al., 2018). Interestingly, treatment with dantrolene improves muscle symptoms such as cramps, myalgia, and muscle weakness in humans or prevents or reduces awake MH crisis in animal models, further supporting the idea of a correlation between anesthesia-induced MH and awake MH (Timmins et al., 2015; Michelucci et al., 2017c). More recently, a correlation between MH and increased levels of blood glucose was observed in humans and in the R163C mouse model for MH (Altamirano et al., 2019; Tamminemi et al., 2020).

Skeletal muscle represents a primary site for insulin-dependent and -independent glucose uptake (DeFronzo 1988; Jessen and Goodyear, 2005). In myofibers, glucose can be used for energy production, if muscle contraction is activated, or stored as glycogen. The mechanism that integrates muscle activity and glucose processing is regulated by changes in intracellular Ca^2+^. Along these lines, a chronic elevation of intracellular Ca^2+^ concentration was reported to correlate with lower expression of the glucose transporter GLUT4 and increased expression of phosphorylated glycogen phosphorylase and glycogen synthase (Park et al., 2009; Tammineni et al., 2020; Uryash et al., 2022). These changes boost glycogen breakdown and reduce glucose uptake, thus promoting insulin resistance and hyperglycemia. Interestingly, treatment with dantrolene improved glucose uptake and tolerance, suggesting that alteration in intracellular Ca^2+^ plays a central role in MH-associated hyperglycemia (Altamirano et al., 2019; Uryash et al., 2022).

### Other RYR1-related myopathies

King–Denborough syndrome (KDS) is characterized by susceptibility to MH, delayed motor development, short stature, cryptorchidism, skeletal abnormalities, and variable dysmorphic features. Resting creatine kinase levels are elevated in some patients, and muscle biopsies show fiber size variation with atrophic type I muscle fibers and absence of cores. KDS can present with either a dominant or a recessive inheritance of mutations in *RYR1* and presents with a high variable penetrance (Isaacs and Badenhorst, 1992; Dowling et al., 2011).

Mutations in *RYR1* have been rarely identified in some other myopathies. For example, three different *RYR1* mutations were identified in calf-predominant distal myopathy, a mild dominant distal myopathy characterized by fatty degeneration of medial gastrocnemius, elevated creatine kinase levels, and the presence of cores in muscle biopsies (Savarese et al., 2020). In contrast to early-onset *RYR1*-related myopathies that affect the medial and anterior thigh compartment, few cases of late-onset axial myopathy have been associated with *RYR1* mutations. These are characterized by paravertebral and posterior thigh involvement (Jungbluth et al., 2009; Løseth et al., 2013). The real incidence of these myopathies is probably largely underestimated, since clinical manifestation typically occurs in old age and can be confused with normal aging dysfunction.

### *CACNA1S-* and *STAC3*-related congenital myopathies

*CACNA1S* mutations are associated with MH and some forms of congenital myopathies (Monnier et al., 1997; Schartner et al., 2017; Mauri et al., 2021). *CACNA1S* mutations identified in MH affect residues located in the S4 voltage-sensing domain, while those identified in congenital myopathies are associated with a decrease in CACNA1S protein expression and impairment of E-C coupling (Maggi et al., 2021; Brugnoni et al., 2022). More severe cases were reported with fetal akinesia or cognitive delay (Yis et al., 2019; Ravenscroft et al., 2021). Recently, homozygous and compound heterozygous mutations in *STAC3* were identified in a rare autosomal recessive congenital myopathy called Native American myopathy, also known as Bailey–Bloch congenital myopathy, a severe myopathy characterized by facial involvement, bone and joint deformities, MH susceptibility, and delayed motor milestones (Horstick et al., 2013; Telegrafi et al., 2017; Grzybowski et al., 2017; Zaharieva et al., 2018). *STAC3* encodes a protein that binds Cav1.1 at triads and is essential for E-C coupling (Horstick et al., 2013; Polster et al., 2016; Perni et al., 2017; Rufenach and Van Petegem, 2021).

## SOCE

SOCE is a ubiquitous Ca^2+^ signaling pathway that, in all cell types, allows regulated entry of Ca^2+^ from the extracellular environment in response to a decrease in ER Ca^2+^ content (Putney, 1986). The existence of this mechanism was initially based on the identification of a Ca^2+^ current activated by depletion of intracellular Ca^2+^ stores called the Ca^2+^ release-activated Ca^2+^ current (I_CRAC_; Hoth and Penner, 1992). 10 yr of intense work led to the discovery of the two main proteins capable of sustaining SOCE: STIM1 (Liou et al., 2005; Roos et al., 2005) and ORAI1 (Feske et al., 2006; Vig et al., 2006; Zhang et al., 2006). Current evidence supports a model in which STIM1 and ORAI1, given their ubiquitous pattern of expression, represent the main contributors to SOCE in eukaryotic cells (Lewis, 2020).

STIM1 is a single-pass transmembrane protein on the ER/SR membrane that functions as a sensor of intraluminal Ca^2+^ levels. This function is mediated by two noncanonical Ca^2+^-binding EF-hand motifs in the N-terminal intraluminal region of STIM1 that, at resting conditions when intracellular Ca^2+^ stores are full, maintain STIM1 in a Ca^2+^-bound dimeric conformation in the ER/SR. ORAI1 is a plasma membrane protein containing four transmembrane helices with both N- and C-terminal regions facing the cytosol (Feske et al., 2006; Vig et al., 2006; Zhang et al., 2006). Six ORAI1 proteins assemble into functional hexameric complexes that form fully functional CRAC channels, allowing Ca^2+^ influx from the extracellular environment to refill intracellular stores (Lewis, 2020). The I_CRAC_ current undergoes fast and slow Ca^2+^-dependent inactivation (CDI) that, by terminating Ca^2+^ entry, prevents the potentially harmful consequences of Ca^2+^ overload for cells (Zweifach and Lewis, 1995a; Zweifach and Lewis, 1995b; Lewis, 2020).

After depletion of intracellular Ca^2+^ stores, the dissociation of Ca^2+^ from STIM1 induces conformational changes resulting in STIM1 adopting a more extended structure and relocating to dedicated ER–plasma membrane junctional sites, where it forms large aggregates. Here, STIM1 binds to phosphoinositide on the plasma membrane and, through a domain in the cytosolic region termed CRAC-activating domain (CAD) or STIM-ORAI–activating region (SOAR), interacts with ORAI1. The site in ORAI1 that mediates the interaction with STIM may require multiple separate domains, one of which has been recently proposed to correspond to a peptide in the cytoplasmic C-terminal extension that follows the fourth transmembrane helix in each of the six ORAI1 monomers (Baraniak et al., 2021).

After the initial identification of STIM1 and ORAI1, additional studies identified a second member of the STIM family (STIM2) and two additional *ORAI1* genes, *ORAI2* and *ORAI3* (Feske et al., 2006). STIM2 shares significant structural homology with STIM1, and the two proteins can form heterodimers (Berna-Erro et al., 2017). Studies on the Ca^2+^ affinities of STIM1 and STIM2 have shown that the latter has a lower affinity for Ca^2+^ than STIM1, supporting a model in which STIM2 is more sensitive to changes in the ER luminal Ca^2+^ and thus may contribute to activate SOCE at front of minimal decreases in Ca^2+^ concentration in the intracellular stores (Brandman et al., 2007; Berna-Erro et al., 2009).

Several isoforms generated by differential splicing of *STIM1* and *STIM2* are expressed in different cells. Three spliced isoforms of *STIM1* have been described that show tissue-specific patterns of expression and different regulatory properties: STIM1L, expressed in skeletal and cardiac muscle and in the brain (Darbellay et al., 2011); STIMB, a neuron-specific isoform (Ramesh et al., 2021); and STIMA, which appears to negatively modulate SOCE (Knapp et al., 2022). Two splice variants of *STIM2* have been identified: STIM 2.1, also called STIM2β, and STIM2.3; the original STIM2 protein is also called STIM2.2 or STIM2α. STIM 2.1 is strong dominant-negative regulator of SOCE, likely because of a short insert of eight amino acids in the CAD/SOAR region that impairs the association of STIM2.1 with ORAI and the transient receptor potential (TRP) channels. Not much is yet known about the STIM2.3 splice variant, which differs from both STIM2.1 and STIM2.2 in the C-terminal region (Miederer et al., 2015; Rana et al., 2015).

The use of alternative translation initiation sites results in expression of two ORAI1 isoforms, ORAI1α and ORAI11β. ORAI11α contains 63 additional amino acids in the N-terminal region, not present in ORAI1β (Desai et al., 2015). As concerns the ORAI channels, while the function of ORAI1 has been intensively studied, much less is known about ORAI2 and ORAI3. *ORAI2* and *ORAI3* share a significant homology to *ORAI1* and, when transfected in cells, form CRAC channels that can be activated by STIM proteins, even if they show some regulatory properties distinct from ORAI1 channels (Emrich et al., 2022). Interestingly, knockout of *Orai2* and *Orai3* in mice and cells has provided evidence that the assembly of heteromeric channels containing ORAI1 with either ORAI2 or ORAI3 results in the negative regulation of ORAI1 (Yoast et al., 2020).

Additional studies have also shown that SOCE, in addition to ORAI1 channels, may occur through the recruitment of additional channels such as the nonselective cation channels of the TRP canonical (TRPC) family (Lee et al., 2010). The contribution of TRPC will result in the activation of a store-operated current (I_SOC_) of divalent and monovalent cations (Lewis, 2020; Emrich et al., 2022).

The existence of two *STIM* and three *ORAI* genes and alternative spliced isoforms, some of which have dominant-negative effects on the I_CRAC_ current, is strongly suggestive of the importance of fine-tuning SOCE-mediated Ca^2+^ signaling to regulate the variety of different functions that operate simultaneously in eukaryotic cells. That SOCE plays signaling functions in addition to refilling the empty Ca^2+^ stores is now supported by several studies that link modification of SOCE function with activation of specific transcription factors and signaling pathways, resulting in the regulation several specific cell functions, including regulation of metabolism. Indeed, SOCE has been shown to contribute to activation of T cell proliferation by triggering calcineurin-mediated activation of the NFAT transcription factor and of stimulation of the PI3K-AKT-mTOR pathways, resulting in upregulation of glucose transporters and glycolytic and mitochondrial enzymes that provide the metabolic support necessary for T cell expansion and activation of adaptive immune functions (Vaeth et al., 2017). Studies with *Orai1*- or *Stim1*/*Stim2*-deficient mice revealed a role of SOCE in regulating fatty acid metabolism in liver, skeletal, and cardiac muscle (Maus et al., 2017). A role of SOCE in regulating mitochondria and glycolysis has also been observed in studies using inducible smooth muscle–specific STIM1 knockout mice (Johnson et al., 2022).

## SOCE regulation, mitochondria, and metabolism

While the mechanisms responsible for SOCE activation are quite well defined, less is known about the mechanisms that terminate CRAC channel activity. Two mechanisms responsible for CDI of CRAC channel activity have been described (Zweifach and Lewis, 1995a; Zweifach and Lewis, 1995b). Fast CDI occurs within tens of milliseconds, and it has been proposed that direct Ca^2+^ binding to ORAI inactivates the channel (Zweifach and Lewis, 1995a); Ca^2+^ and Ca^2+^-calmodulin (CaM) binding to STIM1 (Litjens et al., 2004; Mullins et al., 2009) and ORAI1 have been described to also have a role in fast CDI (Litjens et al., 2004; Srikanth et al., 2010). Interestingly, ORAI1β has a lower fast inactivation rate than ORAI1α, suggesting also that the 63-aa insert present in ORAI1α might be involved in fast inactivation (Parekh, 2017). On the other hand, it has been suggested that a slow, Ca^2+^-dependent conformational change in the STIM1/ORAI1 complex due to store refilling or reversible biochemical changes may be responsible for slow CDI (Parekh, 2017).

Mitochondria, although not directly involved in SOCE activation, can attenuate the slow phase of CDI by lowering cytosolic Ca^2+^ levels in proximity to activated ORAI channels by transporting Ca^2+^ to the mitochondrial matrix through the mitochondrial calcium uniporter (MCU; Parekh 2008; Mammucari et al., 2018). Indeed, if mitochondria are depolarized, Ca^2+^ entry into the mitochondrial matrix is reduced and cytosolic Ca^2+^ remains high, resulting in CRAC channel inactivation. Because fast CDI is not affected by mitochondria depolarization, it has been suggested that mitochondria are involved in regulation of slow CDI (Glitsch et al., 2002). Increases in mitochondrial Ca^2+^ concentration regulate ATP production by enhancing the synthesis of NADH and FADH2 (Rossi et al., 2019b), thus linking ATP production to muscle demand. Moreover, the dynamics of Ca^2+^ transport in and out of mitochondria and the effects of Ca^2+^ on the enzymes that govern ATP synthesis activate additional mechanisms by which mitochondria can modulate SOCE (Muallem, 2007; Ben-Kasus Nissim et al., 2017; Walters and Usachev, 2022).

More recently, however, the role of mitochondria in regulating SOCE by preventing CDI has been challenged by studies based on knockout and knockdown of the MCU. These studies revealed that, against expectations, deletion of the MCU stimulated an increase in SOCE-mediated Ca^2+^ entry, even if CDI of these channels was promoted by MCU deletion (Yoast et al., 2021). Using mathematical simulations, those authors showed that mitochondrial Ca^2+^ transport can act on different pathways to finely regulate Ca^2+^ homeostasis. Altogether, it appears that the role of mitochondria in regulating SOCE must occur through additional mechanisms other than simply removing Ca^2+^ from the cytosol and prolonging SOCE current by delaying inactivation (Yoast et al., 2021).

An additional regulator of SOCE is SARAF, an ER-resident protein that can exert activating and inactivating effects on CRAC channels by interacting with the SOAR domain or the inhibitory domain in STIM1 (Palty et al., 2012; Dagan and Paltry, 2021; Zomot et al., 2021). Several other proteins interact and regulate SOCE, including STIMATE (STIM-activating enhancer; Lopez et al., 2016) and CASQ1, the main Ca^2+^ buffer of the SR (Shin et al., 2003; Zhang et al., 2016).

## SOCE in skeletal muscle

In recent years, it has become evident that also skeletal muscle fibers depend on SOCE mechanism to replenish SR Ca^2+^ reserves (Kurebayashi and Ogawa, 2001; Launikonis and Rios, 2007). In mammalian skeletal muscle, STIM1 and STIM2 are expressed together with some of their alternative spliced isoforms. STIM1L is a longer splice variant of STIM1 that, in the C-terminal region, contains an additional 106 amino acids encoding an actin-binding domain that allows STIM1 to interact with the subcortical actin filaments (Darbellay et al., 2011; Lilliu et al., 2021). This interaction supports the permanent assembly of STIM1L clusters in proximity to ORAI1 channels on the plasma membrane. Based on this stable position, STIM1L was proposed to be mainly responsible for the faster activation kinetics of SOCE observed in skeletal muscle (Darbellay et al., 2011). Nevertheless, it is not clear if this occurs through ORAI1 activation, since STIM1L appears to be less efficient than STIM1 at activating ORAI1, while it shows better interaction and functional activation of TRPC1 and TRPC4 (Dyrda et al., 2020). Indeed, the expression of STIM1L together with TRPC1 and TRPC4 has been reported to be required for fusion and differentiation of myoblasts, and biophysical studies demonstrated that all three proteins are required for optimal SOCE kinetics in myotubes (Antigny et al., 2017; Dyrda et al., 2020). STIM2.1, an alternatively spliced isoform of STIM2, is also expressed in skeletal muscle. The STIM2.1 isoform can assemble with other STIM isoforms, resulting in heterodimers that negatively regulate SOCE. The resulting modulation of Ca^2+^ homeostasis appears to stimulate myogenic differentiation by increasing the Ca^2+^-regulated expression of NFAT4 and MEF2C transcription factors (Kim et al., 2019). Accordingly, muscle-specific *Stim1* knockout mice show a reduction in Ca^2+^-dependent signal transduction pathways involved in muscle growth, thus resulting in growth delay and postnatal lethality (Li et al., 2012).

In adult skeletal muscle, SOCE was found to be activated at triads by single action potentials immediately after Ca^2+^ was released by RYR1 following activation by DHPRs. Detailed studies demonstrated that, despite global SR Ca^2+^ content remaining constant during E-C coupling, Ca^2+^ concentrations at the j-SR drop significantly; it has been proposed that local, but consistent, reduction in SR Ca^2+^ content rapidly activates SOCE well before global SR Ca^2+^ depletion (Launikonis et al., 2009; Koenig et al., 2018; Pearce et al., 2022). Studies on SOCE in skeletal muscle have been also extended to its role in maintaining the SR Ca^2+^ stores following repetitive stimulation of muscle contraction, and especially after intense prolonged activity (Boncompagni et al., 2017; Michelucci et al., 2018; Michelucci et al., 2019; Michelucci et al., 2020; Lilliu et al., 2020). Interestingly, intense exercise was shown to induce the assembly of new intracellular junctions between T tubules and l-SR, where STIM1 and ORAI1 are colocalized. These newly identified structures, named Ca^2+^ entry units (CEUs), are formed by elongations of T tubules that run parallel to the l-SR. CEUs support increased Ca^2+^ entry via ORAI1 and contribute to improve fatigue resistance under continued muscle activity (Michelucci et al., 2019; Michelucci et al., 2020). In contrast to studies in which activation of SOCE was observed only at triads (Cully et al., 2017), CEUs appear to connect ORAI1 on the T tubules with STIM1 on the l-SR (Boncompagni et al., 2017; Michelucci et al., 2020), suggesting that Ca^2+^ entry may occur at different sites in skeletal muscle fibers. To avoid cytosolic Ca^2+^ overload and help preserving energy for muscle contraction and reducing fatigue, STIM1 can be phosphorylated by the 5′AMP-activated kinase (AMPK) during exercise, resulting in a decrease in SOCE, which may avoid cytosolic Ca^2+^ overload, help preserve energy for muscle contraction, and reduce fatigue (Nelson et al., 2019).

Evidence of an additional role of STIM1, but not of ORAI1, in skeletal muscle has recently been observed in response to the high energy demand that occurs during exercise. Experiments with inducible knockout of STIM1 showed that the reduction in muscle mass and exercise capacity following STIM1 ablation was due not only to a direct impact on Ca^2+^ availability for muscle contraction, but also to alterations in muscle metabolism and increase in ER stress/UPR (Wilson et al., 2022). Because these effects were not observed in *Orai1*-deficient mice, they may result from a direct interplay between STIM1 and mitochondria, independent of SOCE. Indeed, STIM1 ablation resulted in an increase in lactate production, mainly due to an increase in glucose use associated with a reduction in the functional activity of Ca^2+^-regulated mitochondrial enzymes such as pyruvate dehydrogenase. In addition, biochemical analysis showed that deletion of STIM1 resulted in the selective dampening of pathways linked to growth, while presumably protein synthesis showed an increase. These apparent opposite findings can be explained by a model in which skeletal muscle of *Stim1*-knockout mice adapts to moderate ER stress levels by slowing growth and augmenting protein quality control mechanisms, resulting in more protein synthesis and turnover with a lower muscle mass (Wilson et al., 2022).

## Altered SOCE mechanism in skeletal muscle fibers and congenital myopathies

Mutations in *ORAI1* and *STIM1* genes can severely affect the SOCE mechanism in different cell types and, consequently, cause a range of human genetic diseases. Patients carrying recessive loss-of function mutations in *STIM1* or *ORAI1* develop a life-threatening immune deficiency, leading to recurrent severe infections accompanied by nonprogressive muscular hypotonia, anhidrotic ectodermal dysplasia, defective dental enamel formation, and mydriasis (McCarl et al., 2009; Fuchs et al., 2012; Wang et al., 2014; Lacruz and Feske, 2015; Silva-Rojas et al., 2020; Conte et al., 2021a).

**Figure 2.**
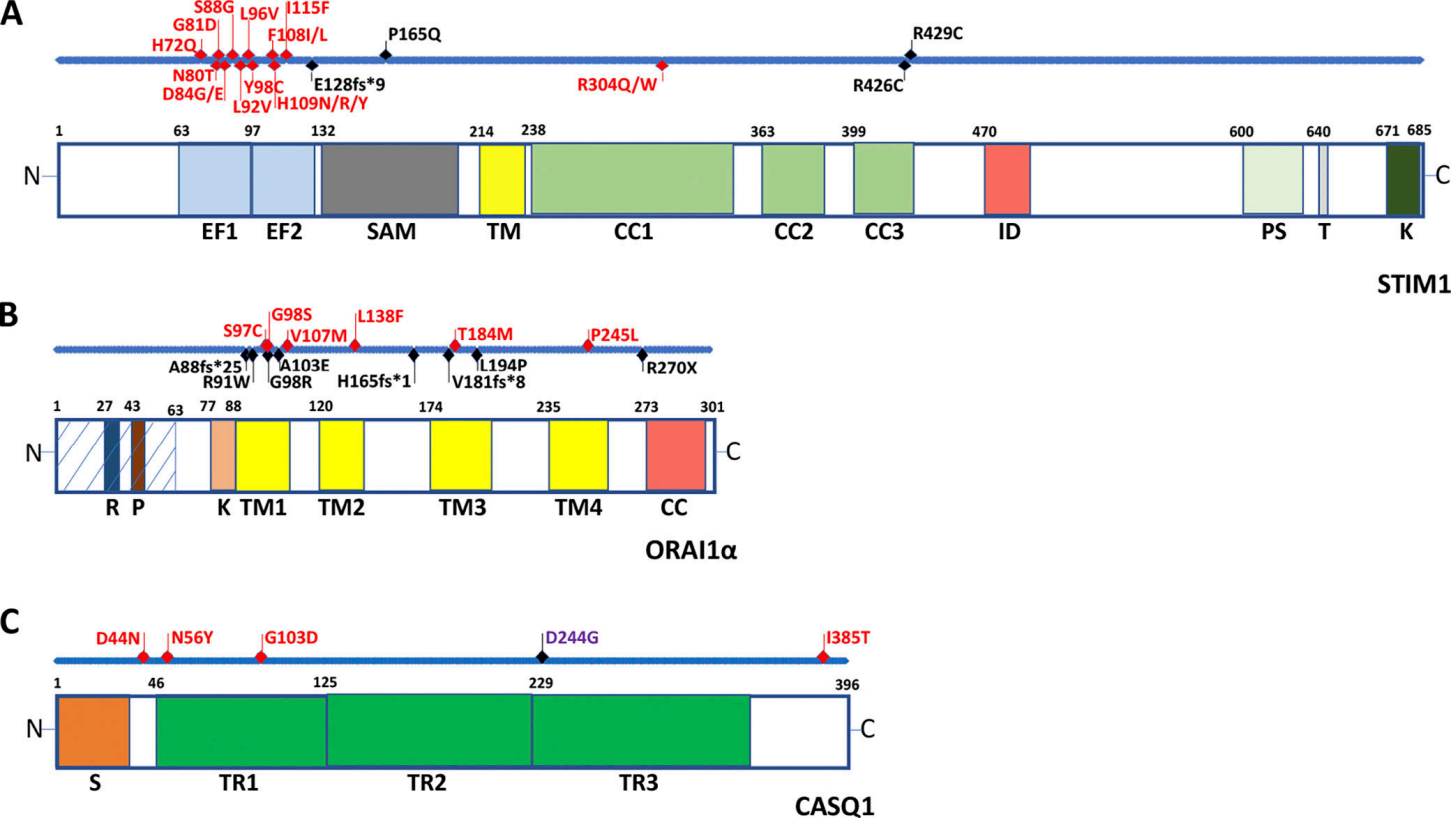
**Schematic representation of STIM1, ORAI1α, and CASQ1 with position of TAM/Stormorken and CRAC channelopathy mutations. (A–C)** Schematic representation of STIM1 (A), ORAI1α (B), and CASQ1 (C) with position of TAM/Stormorken and CRAC channelopathy mutations (depicted in red and in black, respectively) aligned with corresponding protein structural regions. D244G CASQ1 mutation (violet) causes vacuolar aggregate myopathy. The position of the initial aa of each protein domain is indicated. Light blue striped box in the N-terminal portion of ORAI1α indicates the region that is not present in the ORAI1β isoform. For STIM1: CC1/2/3, coiled-coil regions 1/2/3; EF1/2, EF-hand motif 1/2; ID, inhibitory domain; K, lysine-rich region; PS, proline/serine-rich region; S, signal peptide; SAM, sterile α-motif; T, TRIP domain; TM, transmembrane domain; for ORAI1α: CC, coiled-coil domain; P, proline-rich region; R, arginine-rich region; RK, arginine/lysine-rich region; TM 1/2/3/4, transmembrane domain 1/2/3/4; and for CASQ1: S, signal peptide; TR1/2/3, thioredoxin domain 1/2/3.

Recessive loss-of-function mutations in *STIM1* and *ORAI1* genes can result from either frameshift mutations or single missense point mutations that lead to either loss of STIM1 and ORAI1 protein or poorly functional proteins (Fig. 2). As a result, at the functional level, loss-of-function mutations induce a strong reduction or completely abrogate CRAC channel activity, hence the name CRAC channelopathy. Although skeletal muscle is also affected by CRAC channelopathies, the clinically most relevant affected cells are cells of the immune system and especially T lymphocytes (Hoth et al., 2000; Weidinger et al., 2013; Lacruz and Feske, 2015; Silva-Rojas et al., 2020).

Mice knocked out for *Stim1* and *Orai1* die around birth (Gwack et al., 2008; Oh-Hora et al., 2008); in contrast, mice with selective skeletal muscle–specific knockout of *Stim1* and *Orai1* are viable and fertile. Skeletal muscle–specific *Stim1* and *Orai1* knockout mice, however, show reduced muscle mass, reduced muscle contractility and force production, and increased susceptibility to fatigue, which can be explained by alterations in both cytosolic and SR Ca^2+^ levels. At the histological and ultrastructural level, muscles revealed a significant reduction in fiber size with appearance of swollen mitochondria (Stiber et al., 2008; Li et al., 2012; Carrell et al., 2016). Muscle-specific conditional *Orai1* knockout mice show a more significant reduction in type I fibers in soleus muscles that may be explained by alterations in transition from fast to slow myosin expression during perinatal muscle remodeling (Carrell et al., 2016).

Similar alterations in terms of muscle mass, SR Ca^2+^ levels, and resistance to fatigue were also observed in mice carrying the dominant-negative mutation E108Q in ORAI1 (E108Q dn*Orai1* mice; Wei-Lapierre et al., 2013). Nevertheless, these mice, unlike *Stim1* and *Orai1* knockout mice, are viable and have a normal lifespan; despite a reduction in muscle mass and complete loss of SOCE, they do not show significant myopathic alterations at the histological level. According to those authors, the differences between STIM1 knockout mice and E108Q dn*Orai1* mice may be explained by the fact that STIM1 also regulates other intracellular targets such as TRPC, adaptor proteins, ER chaperones, and second messenger enzymes (Wei-Lapierre et al., 2013).

## Gain-of-function mutations in STIM1 and ORAI1: tubular aggregate myopathy (TAM)/Stormorken syndrome

Gain-of-function mutations that result in constitutive and/or overactivation of SOCE are mainly associated with TAM and Stormorken syndrome (Chevessier et al., 2004; Chevessier et al., 2005; Morin et al., 2020). TAM is a rare genetic disease that selectively affects skeletal muscle. At the clinical level, TAM patients present a range of symptoms that may include muscle weakness, myalgia, cramps, and increased creatine kinase levels and exercise intolerance and can start in infancy and worsen over time, although diagnosis at adult age is also reported. Proximal muscles of lower limbs are predominantly affected, but other skeletal muscles can also be affected (Böhm et al., 2013, 2017; Nesin et al., 2014; Endo et al., 2015; Böhm and Laporte, 2018; Silva-Rojas et al., 2020). Patients with gain-of-function mutations in *STIM1* and *ORAI1* may also present with a rare, severe multisystem disorder, Stormorken syndrome, that shares with TAM the skeletal muscle involvement but, in line with the wide cell and tissue expression patterns of *ORAI1* and *STIM1*, is characterized by a variety of additional symptoms that affect other tissues. As a whole, TAM and Stormorken syndrome are actually considered a clinical continuum of manifestations characterized by muscle weakness, myalgia, and cramps, mostly at the level of the lower limbs, that in some patients may be accompanied by a variable range of additional symptoms such as miosis, ichthyosis, thrombocytopenia, short stature, and dyslexia (Stormorken et al., 1985; Misceo et al., 2014; Böhm and Laporte, 2018; Silva-Rojas et al., 2020).

At the histological level, muscle biopsies from TAM/Stormorken patients present a prevalence of type I fibers and a characteristic pattern, predominantly found in type 2 fibers, consisting of an accumulation of highly ordered and packed membrane tubules that appear bright red with the modified Gomori trichrome technique and stain positive with periodic acid–Schiff (PAS) and NADH-tetrazolium reductase, but are negative for succinate dehydrogenase or cytochrome c oxidase staining (Chevessier et al., 2005). When visualized by EM, they appear as single- or double-walled tubules arranged as honeycomb-like structures (Salviati et al., 1985; Schiaffino, 2012; Böhm et al., 2013; Chevessier et al., 2005). These tubular aggregates are positive in immunofluorescence for several SR proteins such as CASQ1, SERCA, triadin, RYR1, and STIM1 (Chevessier et al., 2005; Morin et al., 2020; Silva-Rojas et al., 2020). What leads to the development of tubular aggregates is not clear, although they are likely to represent the final stage of a protective mechanism aimed to prevent muscle hypercontraction and damage caused by altered Ca^2+^ homeostasis that, by inducing protein misfolding and aggregation, causes morphological changes in the SR that ends in formation of tubular aggregates (Chevessier et al., 2004; Schiaffino, 2012; Chevessier et al., 2005; Morin et al., 2020).

Several gain-of-function mutations in *STIM1* have been identified, the majority of which affect different amino acids in the canonical and noncanonical EF-hand motifs (Böhm et al., 2013; Böhm and Laporte, 2018; Fahrner et al., 2018; Morin et al., 2020; Silva-Rojas et al., 2020) or the R304 residue in the coiled-coil domain 1 (CC1) of the protein (Misceo et al., 2014; Nesin et al., 2014; Harris et al., 2017; Peche et al., 2020). In the Ca^2+^-bound state of STIM1, the canonical and noncanonical EF-hand motifs and the sterile α-motif domain are tightly packed together (Enomoto et al., 2020). Mutations in the EF-end motifs essentially alter or disrupt Ca^2+^ binding, resulting in constitutive active SOCE due to domain unfolding (Böhm et al., 2013, Böhm et al., 2017; Sallinger et al., 2020). As observed in muscle cells from mouse models or in myoblasts from patients carrying these mutations, the mutations induce constitutive STIM1 clustering, independently of SR Ca^2+^ depletion, that results in constitutive activation of SOCE, leading to increased Ca^2+^ levels in both the cytosol and the SR (Cordero-Sanchez et al., 2019; Silva-Rojas et al., 2019; Conte et al., 2021b). Studies performed on channels carrying mutations in the noncanonical EF hand motif revealed that constitutive SOCE was also associated with induction of autophagic processes (Sallinger et al., 2020) and mitochondria degeneration (Cordero-Sancez et al., 2019). Studies on in vitro differentiation of myoblasts from patients carrying the *STIM1* L96V mutation showed that these cells displayed reduced multinucleation and alteration in the mitochondrial network, confirming that SOCE also plays a relevant role during development and differentiation (Conte et al., 2021b).

The R304W mutation in *STIM1* has been extensively studied (Nesin et al, 2014; Fahrner et al, 2018; Rathner et al, 2021; Silva-Rojas et al, 2021). This mutation affects one conserved residue in CC1, unlocking the inhibitory state of STIM1 and constitutive SOCE activation (Morin et al., 2020). In addition, the R304W mutation suppresses fast CDI of ORAI, further enhancing chronic Ca^2+^ influx from the extracellular space (Nesin et al., 2014).

Gain-of-function mutations in *ORAI1* were identified in the transmembrane domains. Mutations in M1, M2, and M3 transmembrane domains appear to induce constitutive channel activity, independently of STIM1 activation (Nesin et al., 2014; Endo et al., 2015; Böhm et al., 2017; Garibaldi et al., 2017; Bulla et al., 2019; Peche et al., 2020), while the *ORAI1* P245L mutation in TM4, identified in a patient with a Stormorken-like syndrome, appears to lengthen the duration of the Ca^2+^ entry current, following activation by store depletion, by removing the slow CDI mechanism, while the fast CDI is preserved (Nesin et al., 2014).

Mouse models for gain-of-function mutations in *Stim1* have been generated and found to present with a variable combination of the clinical signs of TAM/Stormorken syndrome in humans. *Stim1*^D84G^ mice mainly present with alteration in platelet activation and bleeding (Grosse et al., 2007). *Stim1*^I115F^ mice have histological and functional alterations in skeletal muscle tissue and display an increased susceptibility to fatigue. They also present with hematological defects with thrombocytopenia and altered differentiation in the myeloid lineage and natural killer cells (Cordero-Sanchez et al., 2019). *Stim1*^R304W^ mice showed defective skeletal muscle function, thrombocytopenia, spleen alteration, anomalies of the eye and skin, altered bone structure, and dysregulation of immune system (Silva-Rojas et al., 2019). Characterization of skeletal muscle of *Stim1*^R304W^ mice revealed evidence of apoptosis, muscle degeneration, and overexpression of ER stress/UPR accompanied by downregulation of RYR1, DHPR, and SERCA1, which could represent a protective mechanism to limit SR Ca^2+^ overload (Silva-Rojas et al, 2021). However, none of these mouse models develops defined tubular aggregates, suggesting that additional factors might be required in mice for development of these structures (Gamage et al., 2018; Silva-Rojas et al., 2019).

## Mutations in CASQ1 and RYR1 in patients with mild forms of TAM

Mutations in *STIM1* and *ORAI* are detected in only a fraction of patients diagnosed TAM/Stormorken syndrome (Morin et al., 2020; Silva-Rojas et al., 2020; Conte et al., 2021a). Studies aimed at identifying additional genes causative of TAM/Stormorken syndrome resulted in the identification of three different causative mutations in the *CASQ1* gene in 11 patients with a TAM diagnosis from 6 unrelated families (Barone et al., 2017; Böhm et al., 2018). CASQ1 is the major Ca^2+^-buffering protein in the SR that, thanks to its ability to bind Ca^2+^ with low affinity and high capacity, plays a key role in providing the high Ca^2+^ storage capacity of the SR necessary for activation of muscle contraction (Rossi et al., 2021). The ability of CASQ1 to bind Ca^2+^ is closely linked to its ability to polymerize and form long ribbonlike structures that allow Ca^2+^ storage within the SR (Park et al., 2003; Sanchez et al., 2012). CASQ1, alone or in combination with junctin and triadin, can regulate RYR1 opening and thus contribute to regulation of Ca^2+^ release (Beard et al., 2002; Gaburjakova et al., 2013; Manno et al., 2017; Rossi et al., 2021). Accordingly, altered polymerization due to mutations in *CASQ1* may affect channel gating, resulting in leaky RYR1 channels. Initial evidence of a possible regulatory role of CASQ1 on the SOCE mechanism was provided by experiments based on knockdown of CASQ1 in muscle fibers that resulted in increased Ca^2+^ entry across the sarcolemma (Zhao et al., 2010) and by experiments indicating that CASQ1 can bind both STIM1 and STIM2 and inhibit SOCE (Shin et al., 2003; Wang et al., 2015; Zhang et al., 2016; Jeong et al., 2021). More recently, *Casq1* knockout mice were shown to have an increased expression of Stim1 and Orai1 associated with enhanced SOCE and preformed CEUs, possibly reflecting a compensatory mechanism to maintain the SR Ca^2+^ at levels sufficient to ensure muscle contraction in the absence of CASQ1 (Michelucci et al., 2020).

At the clinical level, *CASQ1* mutations were detected in patients that reported progressive muscle weakness and exercise-induced myalgia with fatigability predominantly involving the proximal limb muscles. Only one patient reported muscle stiffness. Serum creatine kinase levels were normal in most patients. As expected, given its selective skeletal muscle expression, TAM patients with *CASQ1* mutations presented signs of only a mild myopathy and, except for one patient who reported ichthyosis, no other symptoms of Stormorken syndrome (Barone et al., 2017; Böhm et al., 2018). All three *CASQ1* mutations identified in these TAM patients altered Ca^2+^-dependent polymerization and reduced Ca^2+^ storage content when transfected in cells. Two of the mutants were shown to have lost the ability to inhibit SOCE, although one mutant was still able to inhibit Ca^2+^ influx (Barone et al., 2017).

A recent study reported the identification of two different missense *RYR1* mutations in two unrelated patients with a mild form of myopathy, where the presence of tubular aggregates in muscle fibers was the only alteration observed on histological examination, thus identifying *RYR1* as the fourth causative gene in TAM (Vattemi et al., 2022). The two patients came to medical examination reporting symptoms since childhood and early adulthood, respectively. Only one patient reported significant muscle weakness and presented with myopathic changes in four limbs by needle electromyography. Both patients had a history of persistent increase of serum creatine kinase levels (two- to fourfold the normal values). Interestingly, the two patients complained of suffering muscle stiffness after repetitive movements, a symptom not previously associated with TAM, but they did not report symptoms related to Stormorken syndrome. Therefore, considering the quite mild phenotype reported, patients with TAM associated with *RYR1* mutations may represent the less severe side of the TAM/Stormorken syndrome. At the functional level, both *RYR1* mutations detected in these patients were shown to alter the properties of the RYR1 channels and have been reported as pathogenic and causative for MH susceptibility based on EMHG guidelines (https://www.emhg.org). One of the two mutations has been also found in patients with CCD. The identification of mutations in the *RYR1* gene in TAM patients is of interest, since *RYR1* mutations may represent the genetic cause of myopathy in at least a fraction of the patients affected by TAM that do not carry mutations in *STIM1*, *ORAI1*, or *CASQ1* and therefore represent a new diagnostic target for these patients. Moreover, since the phenotype of both patients is rather mild, it is possible that *RYR1-*related TAM may be currently underdiagnosed in the population.

## Concluding remarks

The development of fast and cost-effective DNA sequencing technologies has tremendously increased our knowledge of the genetic basis of skeletal muscle pathologies. This has provided further evidence that mutations in one gene, as in *RYR1*-related disorders, result in several different myopathies characterized by a spectrum of clinical and histopathological phenotypes. At the same time, as in a mirror image, we have seen an increase in the number of genes that can be associated with the same disease (Jungbluth et al., 2018; Lawal et al., 2018; Garibaldi et al., 2019; Lawal et al., 2020). The recent identification of tubular aggregates, a hallmark of TAM/Stormorken syndrome, in patients carrying *RYR1* mutations represents only the latest evidence of how complex it is to associate genetic data and histopathological patterns (Vattemi et al., 2022).

Although it is easy to understand that mutations in *RYR1* or *CACNA1S* can be found in patients with MH or CCD and that mutations in *STIM1* or *ORAI1* are associated with TAM/Stormorken syndrome, less obvious are the cases where mutations in genes not involved in Ca^2+^-handling pathways, such as *MYH7*, *TTN*, or *MEGF10*, are detected in patients presenting with clinical symptoms and histopathological alterations like those present in *RYR1-*related myopathies. This apparent incongruency can be rationalized by evidence that, at least in some cases, mutations in genes apparently not directly connected with Ca^2+^ signaling may indirectly affect regulation of Ca^2+^ homeostasis, as proposed for *SEPN1* (Chernorudskiy et al., 2020) or *BIN1*, *MTM1*, or *DNM2* (Gómez-Oca et al., 2021). However, more work is needed in this direction to explain how genes not known to affect Ca^2+^ signaling mat induce a myopathy. Future advancements in studying functions, regulatory properties, and even more the network of interactions participated by proteins encoded by causative genes of interest will certainly help answer these questions. In this perspective, a significant contribution can be also provided by the identification of genetic or drug modifiers (Bazrafshan et al., 2021; Volpatti et al., 2020). It can be expected that future investigation will address the identification of the pathogenic pathways activated by different genes, and how these may translate in the development of the different histological findings that characterize these myopathies. A better understanding of these pathogenic mechanisms will improve our knowledge, contribute to the classification of these myopathies, and likely identify novel targets for pharmacological and/or genetic intervention to cure these diseases.
